# Accumulation of essential (copper, iron, zinc) and non-essential (lead, cadmium) heavy metals in Caulerpa racemosa, sea water, and marine sediments of Bintan Island, Indonesia

**DOI:** 10.12688/f1000research.54445.1

**Published:** 2021-07-30

**Authors:** Tengku Said Raza’i, . Thamrin, . Nofrizal, Viktor Amrifo, Hilfi Pardi, Imam Pangestiansyah Putra, Try Febrianto, Aidil Fadhli Ilhamdy

**Affiliations:** 1 Faculty of Marine and Fisheries Sciences, Raja Ali Haji Maritime University, Tanjungpinang, Riau island, 19100, Indonesia; 2 Environmental Science Doctoral Program, Riau University, Pekanbaru, Riau, 19200, Indonesia; 3 Faculty of Fisheries and Marine Sciences, Riau University, Pekanbaru, Riau, 19200, Indonesia; 4 Department of Chemistry Education, Faculty of Teacher Training and Education, Raja Ali Haji Maritime University, Senggarang, Tanjungpinang, Riau Island, 19100, Indonesia

**Keywords:** Heavy metal, Caulerpa racemosa, Bintan Island, Atomic Absorption Spectrophotometry

## Abstract

**Background:** Heavy metals are materials naturally occurring in nature and increase with a rise in human activity. Ex-mining areas and domestic waste from human settlements are sources of heavy metal contamination that enter and pollute water, which then accumulates in various organisms including the *Caulerpa racemosa* community. The accumulation of heavy metals in *C. racemosa* has a wide impact on the food chain in aquatic ecosystems and humans because this alga is a consumptive commodity.

**Methods: **Sampling of *C. racemosa* was carried out at seven sites on Bintan Island, Indonesia covering the eastern (Teluk Bakau, Beralas Pasir, Malang Rapat), northern (Berakit and Pengudang), western (Sakera), and southern parts (Tg. Siambang). Sampling was carried out during different monsoons, and heavy metals in water and sediment samples were measured to determine the heavy metal concentration. Heavy metals were analyzed by a spectrophotometric method using Atomic Absorption Spectrophotometry.

**Results:** The results showed that heavy metal concentrations fluctuate according to changes in the wind season, which carry currents and spread pollutants into the water. The concentration of metal in the water is also from anthropogenic activities. The heavy metal content of cadmium (Cd), lead (Pb), copper (Cu), iron (Fe), and zinc (Zn) in *C. racemosa* is high in locations close to settlements. Meanwhile, in seawater samples, Fe and Zn metals have the highest concentrations compared to others.

**Conclusions:** Ex-bauxite mines are a source of Fe and Zn metal contamination in the environment, especially at Tg. Siambang. The levels of these heavy metals in the sediment are also high, as surface particle deposits accumulate at the bottom of the sediment. In general, the levels of heavy metals Cd, Pb, Cu, Fe, and Zn increase in the northern monsoon because the dynamics of the water transport greater heavy metal pollution.

## Introduction

Heavy metals are materials naturally contained in nature. These metals increase along with a rise in human activity and become a threat to environmental pollution. The increase in heavy metals is caused by mining, ^1^ agriculture and industry ^2^ activities, as well as anthropogenic processes from residential and household domestic waste. ^3, 4^ Therefore, increased coastal activity leads to a rise in the content of these metals up to the quality standard threshold. ^5^

Heavy metal particles that accumulate in the environment affect aquatic ecosystems. The contamination is distributed in the water column and accumulates in organisms over a prolonged period, affecting the food chain. ^5^ Briffa *et al.* (2020) stated that heavy metal contamination in organisms affect biological functions and cause various toxic effects in the long term. ^6^ According to Liu *et al*. (2020) and Apiratikul *et al.* (2004), these metals are generally dominated by cadmium (Cd), lead (Pb), copper (Cu), iron (Fe), and zinc (Zn). ^1, 7– 9^

Cadmium (Cd) and lead (Pb) are non-essential heavy metals whose function in the human body are unknown. These metals are categorized as carcinogenic (or mixed) agents for humans by the International Agency for Research on Cancer. Exposure to Cd and Pb causes cancer or infection in human organs such as the urinary tract, reproductive tract, central nervous system, respiratory system, ^10, 11^ they cause kidney problems, and increase blood pressure. ^11^ The content of these heavy metals are measured in blood, urine, hair, nail, and saliva samples and are characterized by low levels of excretion. ^12^ Furthermore, excess exposure to Cd and Pb is lethal when entering mammalian cells and they accumulate high concentrations in the cytoplasmic space and nucleus. ^13, 14^ The maximum limits of Cd and Pb metals in the human body are 0.005 mg/L and 0.015 mg/L, respectively. ^15^ Therefore, due to the high level of toxicity of heavy metals, serious monitoring of the accumulation of Cd and Pb is needed.

Meanwhile, copper (Cu), zinc (Zn), and iron (Fe) are essential heavy metals, with a certain amount needed in the human body. However, when the amount exceeds the maximum limit, it becomes poisonous and very dangerous to health. Cu is considered a carcinogen metal because it causes damage to DNA. Meanwhile, Cu ^2+^ interacts with lipid hydroperoxides to form malondialdehyde and 4-hydroxynonenal, which are considered carcinogens and cause DNA and tissue damage. ^16, 17^ Excessive accumulation of Zn metal causes depletion of Cu ^2+^ in cells and decreases superoxide dismutase (SOD) and cytochrome C oxidase levels by increasing cholesterol levels. Furthermore, it causes cardiac dysfunction with impaired iron mobilization, inhibits the enzymes SOD, peroxidase, and catalase, thereby rapidly increasing the concentration of superoxide free radicals and oxidative stress. Excess Zn also causes severe damage to the cell wall and DNA while increasing gene mutations. ^18^ Fe metal is also one of the important elements in the body with a tightly controlled concentration. Excess Fe is caused by several conditions such as frequent transfusions, exploitation of iron consumption (as a supplement), and chronic hepatitis. It triggers the formation of free radicals, thereby causing severe complications such as mental retardation, Alzheimer's, multiple sclerosis, reproductive system dysfunction, heart problems, liver cirrhosis, liver cancer, hepatitis, and metabolic dysfunction. ^19^ The maximum limits for the essential heavy metals Cu, Zn, and Fe in the human body are 1.3 mg/L, 3 mg/L, 0.2 mg/L, respectively. ^15^ Therefore, it is necessary to monitor the concentration of these heavy metals to prevent them from passing the accumulated threshold.

Aquatic ecosystems consist of various types of biota including the macroalgae community such as *Caulerpa racemosa.* Heavy metal pollution in the aquatic environment also impacts the accumulation of these metals in macroalgae. ^7^ *Caulerpa* sp. is a type of algae that is able to absorb Cd, Pb, Cu, Fe and Zn metals in water. ^20^ The absorption ability of Pb, ^21^ Cd 30 g/L, ^22^ Zn and Cu 10 mg/L, ^23^ and Fe 7.63 mg/L ^24^ in water is 0.35 mg/kg. This proves that *C. racemosa* has the ability to bioaccumulate heavy metals in marine waters.

On the other hand, *C. racemosa* has consumptive benefits for coastal communities, especially as a traditional food ingredient. Khandaker *et al.* (2021) stated that the accumulation of heavy metals in these organisms have the potential to cause various adverse health effects. ^25^ The content of heavy metals contained in *C. racemosa* in Bintan Island, Indonesia waters is unknown, therefore, it is important to carry out this research in order to determine the impact of its exposure to surrounding communities.

## Methods

**Figure 1.**
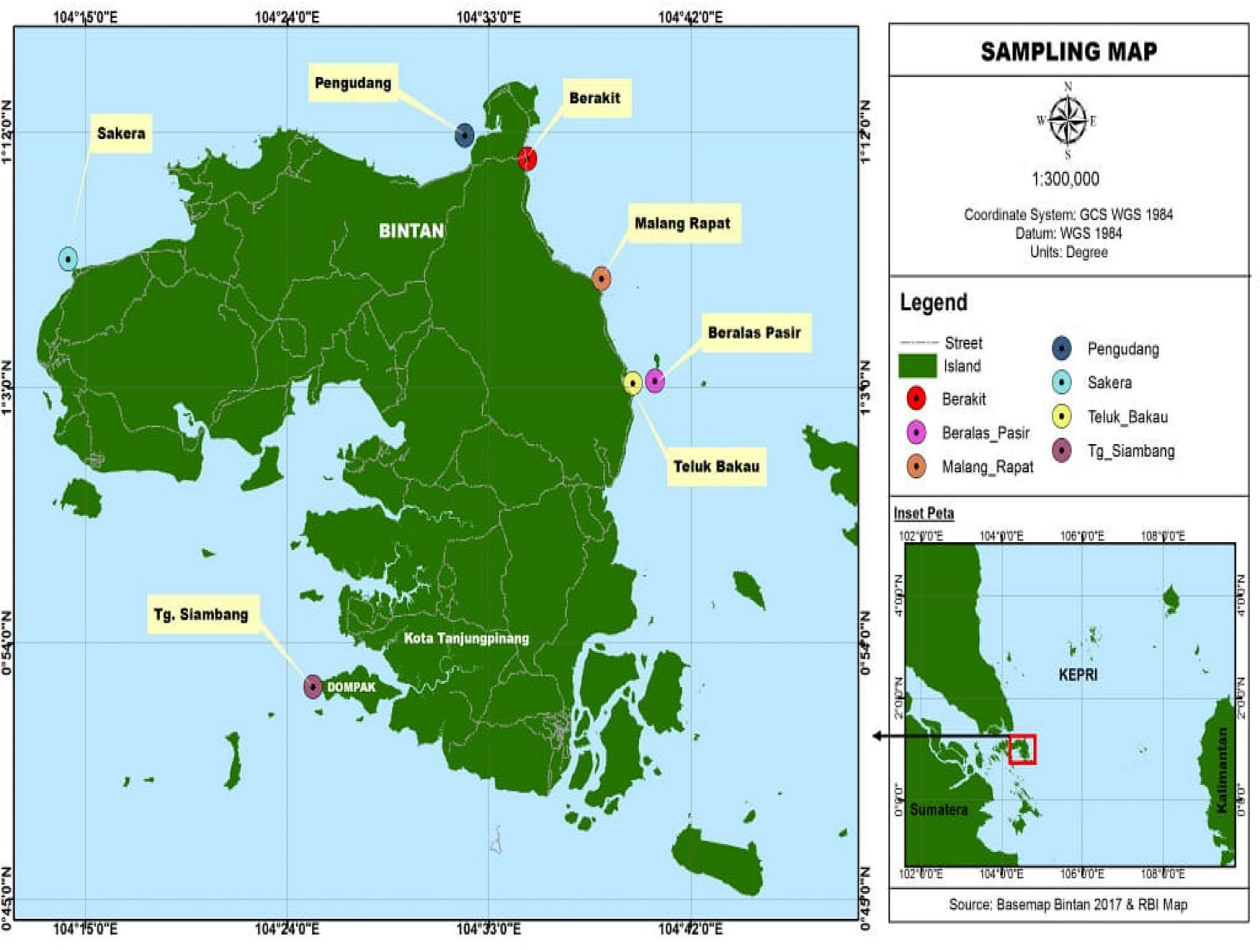
Map of research locations and field data collection, This figure has been reproduced with permission from Apriadi *et al*., (2018). ^58^

Data were collected from seven sites on Bintan Island, Indonesia covering the eastern (Teluk Bakau, Beralas Pasir, Malang Rapat), the northern (Berakit and Pengudang), the western (Sakera), and the southern part (Tg. Siambang). In addition, data was also taken based on seasonal differences to determine the effect of seasonal changes on the existing conditions of *C. racemosa* on the coast of Bintan Island. The difference in seasons is taken to refer to the difference in the direction of the monsoon, namely the northerly, easterly, southerly and westerly monsoon. Figure 1 shows the distribution of research sites on Bintan Island.

Sampling of *C. racemosa* was carried out using a scuba set (Amscud), 50 × 50 cm ^2^ transect plot, underwater camera (Canon D30 82 '/25m rated depth), GPS (Garmin GPSMAP 78S), Oven (Memmert UN 55), analytical scales (Kern ABJ 220, accuracy level of 0.001 g), Van Dorn sampler, sediment shovel, mortar, aluminium foil, plastic samples, label paper, and boxes. Heavy metal levels were tested by the wet digestion method using a solution of nitrite acid (HNO _3_), sulfuric acid (H _2_SO _4_), hydrogen peroxide (H _2_O _2_), and hydrochloric acid (HCl). ^20^ Samples were analyzed using Atomic Abstraction Spectrophotometry (AAS) using the Shimadzu AA-7000. ^26^

### Caulerpa racemosa sampling

The sample preparation process was carried out by weighing 5 grams of the dry weight of the *C. racemosa* sample, which had previously been cleaned and dried in an oven at 60°C for 24 hours. ^27, 28^ The sample was then crushed with a mortar until homogenous before adding a HNO _3_ solution and 20 ml of HCl. Furthermore, the solution was placed in a water heater until it reacted and was digested. It was then filtered with Whatman 42 filter paper to determine the readings of Cd, Pb, Cu, Fe, Zn on the AAS tool.

### Water and sediment sampling

Water and sediment sampling for heavy metal analysis refers to the digestion method. ^29^ Approximately three liters of seawater sample were taken with a Van Dorn sampler and then concentrated by baking at 80°C until the volume reduced to 50 ml. Furthermore, 4 ml of H _2_SO _4_, and 10 ml of H _2_O _2_ were added to the sample and heated until the oxidation was complete. After cooling the sample, it was filtered with 0.45 μm Whatman 42 filter paper and distilled water was added until it reached a volume of 50 ml.

The sediment taken in the field was weighed, and as much as 30 g was dried in an oven at 105°C. In addition, approximately 1 g of the sediment was added to 5 ml of HNO _3_ and heated at 80°C until the volume changed (20–30 ml). The process of adding HNO _3_ was repeated and it was reheated, after which it was filtered with 0.45 μm Whatman filter paper and distilled water was added until it reached a volume of 50 ml.

### Statistical analysis

The results of the metal content readings of Cd, Pb, Cu, Fe, Zn were analyzed using IBM SPSS Statistics 22 software with one-way Anova analysis (p <0.05) to determine the concentration of heavy metals at each site based on the season. Furthermore, the level of correlation between metals was also analyzed to determine the influence of each metal being measured.

## Results and discussion

### Heavy metals in *Caulerpa racemosa* samples

Heavy metal analysis was distinguished by season to determine the content in *C. racemosa* and to explain the effect of the season on the water. Those heavy metals measured in the *C. racemosa* sample consisted of Cd, Pb, Cu, Fe, and Zn in micrograms per gram (μg/g), with the analysis results shown in Table 1. The concentration of the metals Cd, Pb, Cu, Fe, and Zn were in the range of 0.14–4.83 μg/g, 1.07–47.95 μg/g, 1.14–8.14 μg/g, 62.4–609.7 μg/g, and 12.7–76.5 μg/g with an average concentration of 0.95 ± 1.15 μg/g, 14.17 ± 10.98 μg/g, 3.07 ± 2.16 μg/g, 220.58 ± 175.16 μg/g, and 35.25 ± 16.54 μg/g, respectively.

**Table 1.** Average concentration of heavy metal (μg/g) of Cd, Pb, Cu, Fe, and Zn in the *Caulerpa racemosa* during Northerly–Westerly monsoon in Bintan Island coastal water. Cd = cadmium, Pb = lead, Cu = copper, Fe = iron, Zn = zinc.

Season	Location	Cd	Pb	Cu	Fe	Zn
Northerly monsoon	Malang Rapat	0.21±0.31	13.07±6.38	3.61±0.69	162.7±31.16	33.1±30.69
	Teluk Bakau	0.70±0.36	25.53±4.31	3.76±1.31	102.7±22.46	56.1±7.42
	Beralas Pasir	0.14±0.38	21.67±3.99	2.49±1.28	147.3±22.84	76.5±25.85
	Berakit	0.81±0.073	16.93±4.18	1.14±1.35	120.3±19.54	66.0±21.35
	Pengudang	0.78±2.29	13.72±17.86	3.7±2.9	165.7±22.54	27.4±25.26
	Sakera	0.92±2.18	22.01±14.7	1.7±2.94	474.18±19.17	30.9±20.95
	Tg. Siambang	4.83±2.45	47.95±20.03	7.43±2.47	609.7±27.98	72.8±17.84
	**Average**	**1.20±1.63**	**22.98±11.92**	**3.40±2.05**	**254.65±201.36**	**51.12±22.94**
Easterly monsoon	Malang Rapat	0.17±0.18	8.34±1.42	2.92±0.51	157.65±24.0	37.4±10.92
	Teluk Bakau	0.52±0.14	6.82±3.93	2.08±0.45	122.4±9.7	27.8±10.9
	Beralas Pasir	0.41±0.21	9.67±2.46	1.98±0.51	111.8±27.42	49.6±10.53
	Berakit	0.69±0.22	14.60±2.51	1.24±0.49	103.0±23.51	39.5±5.65
	Pengudang	0.82±1.68	12.06±6.22	2.23±3.52	154.3±10.2	28.54±3.76
	Sakera	1.12±1.6	17.09±5.67	1.85±3.36	495.8±16.85	31.6±5.18
	Tg. Siambang	3.88±1.87	24.43±6.53	8.14±3.43	537.4±25.5	24.11±9.64
	**Average**	**1.09±1.27**	**13.29±6.07**	**2.92±2.35**	**240.34±190.19**	**34.08±8.73**
Southerly monsoon	Malang Rapat	0.32±0.26	12.93±2.71	1.65±0.31	118.3±10.5	43.4±11.8
	Teluk Bakau	0.15±0.27	9.04±4.7	2.17±0.28	97.88±9.86	26.1±0.28
	Beralas Pasir	0.67±0.08	14.25±5.08	1.62±0.25	112.4±4.25	26.7±7.96
	Berakit	0.52±0.11	18.43±8.37	1.75±1.28	116.7±17.5	26.3±13.2
	Pengudang	0.67±0.93	8.32±14.01	2.11±3.02	108.2±14.59	12.7±20.33
	Sakera	0.45±0.92	24.93±9.3	4.13±2.62	298.6±17.39	39.1±9.88
	Tg. Siambang	2.17±0.93	36.18±17.56	8.05±2.91	391.9±17.2	52.7±9.93
	**Average**	**0.71±0.67**	**17.73±9.93**	**3.07±2.36**	**177.71±117.76**	**33.48±14.34**
Westerly monsoon	Malang Rapat	0.42±0.11	1.07±0.82	2.92±0.51	62.4±25.36	38.7±10.8
	Teluk Bakau	0.19±0.07	1.53±0.57	2.08±0.45	111.8±9.69	17.2±6.79
	Beralas Pasir	0.32±0.26	2.67±0.49	1.98±0.51	97.1±11.23	29.8±6.32
	Berakit	0.23±0.24	1.93±0.15	1.24±0.49	93.5±26.23	19.1±1.66
	Pengudang	0.72±1.43	1.72±3.51	2.23±3.52	76.1±25.14	18.6±3.35
	Sakera	0.56±1.41	2.01±3.24	1.85±3.36	493.7±17.6	21.7±1.86
	Tg. Siambang	3.12±1.3	7.95±5.74	8.14±2.96	532.8±18.35	25.3±6.04
	**Average**	**0.79±1.04**	**2.70±2.37**	**2.92±2.3**	**209.63±208.31**	**24.34±7.69**

* The residential areas: Ml. Rapat, Tl. Bakau, B. Pasir, Pengudang, Sakera.

** The ex-bauxsite mine: Tg. Siambang.

Research by Khaled *et al.* (2014) obtained the results of the content of several metals in macroalgae, namely Cd (0.26 μg/g), Cu (6.0 μg/g), Fe (177.79 μg/g), Pb (41.16 μg/g) and Zn (59.13 μg/g). ^30^ Yulianto *et al.* (2018) showed that the concentration of Cd metal in seaweed ranged from 0.018 to 0.87 μg/g. ^31^ Meanwhile, research by Llanos and Dalawampu (2017) on a *C. racemosa* sample stated that the concentration of heavy metals such as Cd, Pb, and Zn was in the range of 0.21–0.29 μg/g, 2.61–7.23 μg/g and 6.03–7.23 μg/g, respectively. ^32^ Intawongse *et al.* (2018) research in *C. racemosa* obtained metal concentrations of Pb (0.43 μg/g), Cd (0.79 μg/g), Cu (1.55 μg/g), Fe (311 μg/g), and Zn (13.4 μg/g). ^33^ The results of the above studies indicate that metal levels in sea water fluctuate according to the conditions, with the concentrations influenced by other effects, especially anthropogenic activities.

Table 1 shows an increasing trend of heavy metal concentrations in residential areas and as a result of former bauxite mining activities. As is shown from seven observations, the waters of Malang Rapat, Teluk Bakau, Berakit, Pengudang, and Sakera. The observation station in Tg Siambang waters is a former bauxite mining area whose effects are now affecting the surrounding aquatic environment.

The metal concentrations of Cd, Pb, Cu, and Fe in the ex-mining area have a higher value than in other areas. For example, the concentration of Cd in ex-mining areas is between 2.17–4.83 μg/g, while in other areas, it is lower between 0.14–1.12 μg/g. Similarly, the levels of Pb, Cu, and Fe increased along with a rise in the intensity of community activities.

Rehman *et al.* (2013) compared the levels of several metals in residential areas and found that the Zn, Fe, Cu and Pb content reached 199.94 μg/g, 195.62 μg/g, 9.18 μg/g, and 13.09 μg/g, respectively. ^34^ According to Vongdala *et al.* (2019) and Agbeshie *et al.*, the concentrations of Fe, Pb, Zn, and Cd at domestic waste disposal sites tend to experience a significant increase. ^35, 36^ Kinuthia *et al.* (2020) stated that an increase in heavy metal concentration positively correlates with the intensity of residential spots. ^37^ This is in line with the results of the study, which identified an increase in the levels of heavy metals Cd, Pb, Cu, Fe, and Zn. Furthermore, this activity causes metal accumulation in *C. racemosa* to be higher with a greater effect.

**Figure 2.**
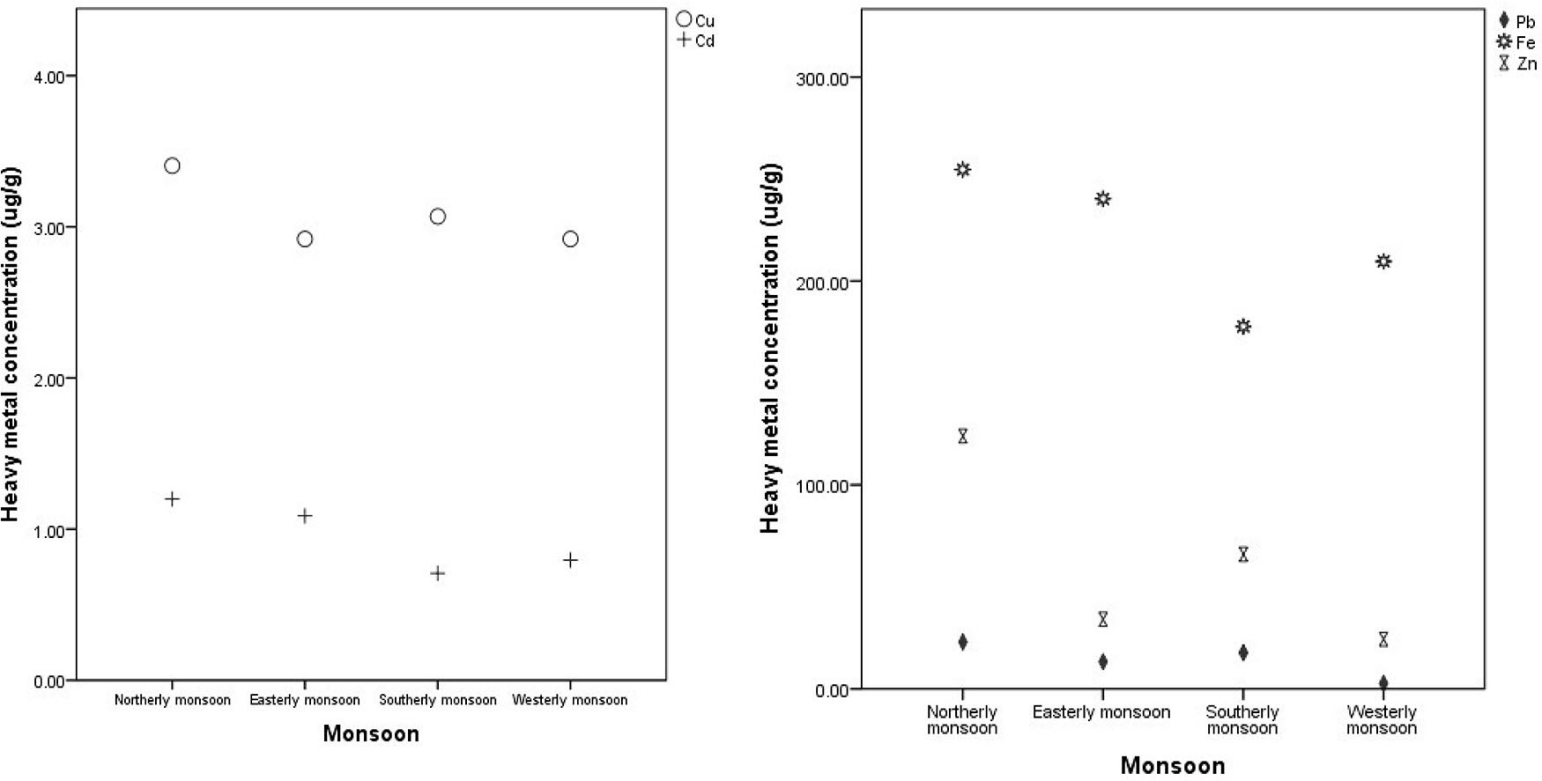
Comparative of heavy metal concentration on Northerly–Westerly monsoon in Bintan coastal water.

Heavy metal concentrations in *C. racemosa* samples were also differentiated according to the wind season, as shown in Table 1 and Figure 2. The aim was to determine the effect of the season on changes in the concentration of heavy metals that accumulated in *C. racemosa.* The concentration of Cd, Pb, Cu, and Fe metals increased in seasons with high wave dynamics and currents (Northerly monsoon). The heavy metals that accumulate in *C. racemosa* are lower in the east-west monsoon (Easterly–Westerly monsoon) than in the north (Northerly monsoon). The Cd metal content in the east-west monsoon, namely 0.71 ± 0.67 and 1.09 ± 1.27 μg/g increased to 1.20 ± 1.63 μg/g in the northern monsoon. Furthermore, the concentration of Pb in the east-west monsoon, namely 2.70 ± 2.37 and 13.29 ± 6.07 μg/g increased in the northern monsoon to 22.98 ± 11.92 μg/g. Additionally, the concentration of Cu in the east-west monsoon was 2.92 ± 2.3 μg/g compared to 3.40 ± 2.05 μg/g in the northern monsoon. Fe content in the east-west monsoon, namely 209.63 ±208.31 and 240.34 ± 190.19 μg/g increased to 254.65 ± 201.36 μg/g in the north monsoon. Zn metal in the east-west monsoon increased from 24.34 ± 7.69 and 34.08 ± 8.73 μg/g to 51.12 ± 22.94 μg/g in the north monsoon. These results indicate that all the tested metals increased in the northern season.

Talwar *et al.* (2014) carried out research related to differences in heavy metal concentrations by season. The results showed that Pb, Cr, and Cu tend to increase during the rainy season by 0.03–0.8 ppm, 0.01–0.4 ppm, and 0.02–0.7 ppm, respectively. ^38^ According to research by Mondol *et al.* (2011), the highest concentration of heavy metals in marine plants is during the wet season, where Pb, Cr, and Cu metals range from 6.18 to 14.91 μg/g, 19.65–19.83 μg/g, and 23.88–33.16 μg/g. ^30^ Zhang *et al*. (2017) stated that the rainy season has the highest effect on the increase in heavy metals. ^39^ This condition is similar to the results found at this research location, where the concentration of heavy metals tends to increase in the northern monsoon due to the effect of current and wave dynamics, which are the driving force for marine dynamics that carry and spread pollutants in waters. ^40^

Koropitan and Cordova (2017) stated that flow is a component of heavy metal transport from a river transported into open water, thereby increasing its value in the aquatic ecosystem. ^41^ Budiyanto and Lestari (2017) examined the difference between metals in the eastern (March) and the southern seasons (June). ^42^ The results showed an increase in Cd, Cu, Pb and Zn metals from 1.18 μg/g to 1.74 μg/g, 94.8 μg/g to 157.0 μg/g, 41.7 μg/g to 89.4 μg/g, and 503.0 μg/g to 1270 μg/g, respectively. These results clearly show that changes in environmental conditions, especially wind direction, dynamics of currents and waves, as well as season, were factors that affected heavy metal concentrations in nature.

The one-way Anova test ( Table 2) showed that the concentration of Pb in *C. racemosa* in the northern monsoon was significantly different compared to other seasons (f = 0.051). Meanwhile, the Cu, Fe, Zn and Cd results were not significantly different between seasons with f values of 0.722, 0.481, 0.121, and 0.493, respectively. Therefore, it can be concluded that the fluctuating season conditions do not significantly affect changes in metal concentrations. The levels of correlation between Cd, Pb, Cu, Fe, and Zn metals are shown in Table 3.

**Table 2.** Anova test of heavy metal concentration on *Caulerpa racemosa* in different monsoon.

Metals		Sum of Squares	df	Mean Square	F	Sig.
Cd	Between Groups	1.146	3	0.382	0.263	0.851
	Within Groups	34.811	24	1.450		
	Total	35.958	27			
Pb	Between Groups	1559.008	3	519.669	7.336	0.001
	Within Groups	1700.020	24	70.834		
	Total	3259.028	27			
Cu	Between Groups	1.095	3	0.365	0.070	0.975
	Within Groups	125.409	24	5.225		
	Total	126.504	27			
Fe	Between Groups	24562.954	3	8187.651	0.244	0.864
	Within Groups	803898.015	24	33495.751		
	Total	828460.969	27			
Zn	Between Groups	42365.448	3	14121.816	1.247	0.315
	Within Groups	271682.633	24	11320.110		
	Total	314048.081	27			

**Table 3.** Correlation of heavy metal in *Caulerpa racemosa* Northerly–Westerly monsoon in Bintan Island coastal water. Cd = cadmium, Pb = lead, Cu = copper, Fe = iron, Zn = zinc.

Heavy Metal	Cd	Pb	Cu	Fe	Zn
Cd	1	-	-	-	-
Pb	0.631 **	1	-	-	-
Cu	0.835 **	0.571 **	1	-	-
Fe	0.77 **	0.526 **	0.651 **	1	-
Zn	−0.059	0.241	0.052	−0.158	1

** Correlation is significant at the 0.01 level (2-tailed).

The results of metal analysis in *C. racemosa* showed a strong correlation level in Pb with Cd, Cu with Cd, Fe with Cd, Cu with Pb, Fe with Pb, Zn with Pb, Fe with Cu, and Zn with Cu. Research by Wang and Liu (2004) found a correlation between Pb and Cd, Cu with Pb, and Zn with Pb metals. ^43^ Furthermore, research by Khaled *et al.* (2014) showed a positive correlation value between metals Cu and Cd, Fe with Cd, metal Cd with Pb, metal Cu with Fe, Cu with Pb, Cu with Zn, and Fe with Pb. ^44^ Harikumar *et al.* (2010) also obtained a positive correlation value between heavy metals Cu and Pb. ^45^ Mourad and El-Azim's research (2019) on macroalgae showed a positive correlation between Cu and Cd, Cu with Pb, Fe with Pb, Zn with Pb, Fe with Cu, and Zn with Cu. ^46^ The concentrations of metals in *C. racemosa* correlate with each other, which indicates that an increase in the levels of one metal affects others.

### Heavy metals in water samples

The concentration of heavy metals in the tested water samples fluctuated according to seasonal conditions. It was found that the highest average concentrations of Cd, Pb, Cu and Fe were in the eastern monsoon, namely 0.0013 ± 0.00056 mg/l, 0.0010 ± 0.00048 mg/l, 0.0010 ± 0.00074 mg/l, and 0.0013 ± 0.00092 mg/l, respectively. Meanwhile, Zn metal was the highest in the western monsoon with an average of 0.0240 ± 0.0054 mg/l, as shown in Table 4.

**Table 4.** Average concentration of heavy metal (mg/l) of Cd, Pb, Cu, Fe, and Zn in the water during Northerly–Westerly monsoon in Bintan Island coastal water. Cd = cadmium, Pb = lead, Cu = copper, Fe = iron, Zn = zinc.

Season	Location	Cd	Pb	Cu	Fe	Zn
*Northerly monsoon*	Malang Rapat	0.0011±0.0001	0.0011±0.0005	0.0005±0.0003	0.0005±0.0002	0.0131±0.012
	Teluk Bakau	0.0009±0.0001	0.0003±0.0006	0.0001±0.0007	0.0006±0.0009	0.0353±0.014
	Beralas Pasir	0.001±0.0001	0.0013±0.0006	0.0008±0.0004	0.0001±0.0009	0.0131±0.002
	Berakit	0.0011±0.00015	0.0015±0.0007	0.0016±0.0005	0.0019±0.001	0.0094±0.004
	Pengudang	0.0009±0.0002	0.0003±0.0006	0.0007±0.0006	0.0003±0.001	0.0148±0.002
	Sakera	0.0012±0.0001	0.0002±0.0005	0.0017±0.0006	0.0023±0.0006	0.0174±0.002
	Tg. Siambang	0.0013±0.0006	0.0013±0.0004	0.0005±0.0002	0.001±0.0003	0.013±0.005
	***Average***	**0.0011** **±0.00015**	**0.0009** **±0.00056**	**0.0008** **±0.00059**	**0.0010** **±0.00085**	**0.0166** **±0.0085**
*Easterly monsoon*	Malang Rapat	0.0023±0.0008	0.0017±0.0006	0.0004±0.0002	0.0007±0.0003	0.0231±0.01
	Teluk Bakau	0.0007±0.0002	0.0004±0.0004	0.0005±0.0007	0.0009±0.001	0.0393±0.01
	Beralas Pasir	0.0011±0.0001	0.0012±0.0002	0.0009±0.0006	0.0003±0.001	0.0192±0.004
	Berakit	0.0008±0.0004	0.0013±0.0004	0.0019±0.0008	0.0023±0.001	0.0112±0.008
	Pengudang	0.001±0.0003	0.0009±0.0004	0.0006±0.0009	0.0006±0.001	0.0159±0.005
	Sakera	0.0017±0.0002	0.0004±0.0004	0.0023±0.0008	0.0027±0.0007	0.0271±0.004
	Tg. Siambang	0.0014±0.0002	0.0012±0.0001	0.0007±0.0002	0.0016±0.0003	0.019±0.002
	***Average***	**0.0013** **±0.00056**	**0.0010** **±0.00048**	**0.0010** **±0.00074**	**0.0013** **±0.00092**	**0.0221** **±0.0091**
*Southerly monsoon*	Malang Rapat	0.0018±0.0006	0.0013±0.0003	0.0005±0.0002	0.0009±0.0002	0.0167±0.006
	Teluk Bakau	0.0005±0.0002	0.0006±0.0003	0.0002±0.0002	0.0006±0.0001	0.0294±0.005
	Beralas Pasir	0.0009±0.0002	0.0012±0.0002	0.0007±0.0001	0.0004±0.0001	0.0183±0.0016
	Berakit	0.0005±0.0004	0.0009±0.0003	0.0004±0.0007	0.0007±0.0003	0.0211±0.0067
	Pengudang	0.0009±0.0009	0.0008±0.0004	0.0007±0.0007	0.0005±0.0003	0.0183±0.0069
	Sakera	0.0001±0.0009	0.0003±0.0004	0.0018±0.0007	0.0011±0.0002	0.0311±0.0058
	Tg. Siambang	0.002±0.0006	0.0012±0.0001	0.0005±0.0001	0.0012±0.0003	0.0201±0.0016
	***Average***	**0.0010** **±0.0007**	**0.0009** **±0.00037**	**0.0007** **±0.00052**	**0.0008** **±0.0003**	**0.0221** **±0.0057**
*Westerly monsoon*	Malang Rapat	0.0009±0.0002	0.0011±0.0002	0.0007±0.0001	0.0006±0.0002	0.0233±0.0019
	Teluk Bakau	0.0007±0.0001	0.0012±0.0003	0.0004±0.0001	0.0007±0.0002	0.0211±0.0072
	Beralas Pasir	0.0004±0.0002	0.0007±0.0001	0.0006±0.0001	0.0003±0.0001	0.0194±0.0079
	Berakit	0.0005±0.0003	0.0005±0.0005	0.0004±0.0002	0.0004±0.0004	0.0328±0.0074
	Pengudang	0.0008±0.0003	0.0004±0.0002	0.0006±0.0002	0.0005±0.0003	0.0188±0.0057
	Sakera	0.0002±0.0001	0.0005±0.0001	0.0002±0.0001	0.0012±0.0002	0.0301±0.0041
	Tg. Siambang	0.0005±0.0002	0.0008±0.0006	0.0004±0.0001	0.0011±0.0002	0.0222±0.0013
	***Average***	**0.0006** **±0.00024**	**0.0007** **±0.00031**	**0.0005** **±0.00017**	**0.0007** **±0.00034**	**0.0240±0.0054**

* The residential areas: Malang Rapat, Teluk Bakau, Beralas Pasir, Pengudang, Sakera.

** The ex-bauxsite mine: Tg. Siambang.

Several studies stated that in marine waters, Pb metal concentrations ranged from 0.0059–1.07 mg/l, ^47^ Cadmium (Cd) ranged from 0.01–0.03 mg/l, ^48^ Cu ranged from 0.0472–0.0725 mg/l, Zn ranged from 32.59–45.79 mg/l, ^49^ and Fe ranged from 0.17–0.45 mg/l. ^50^ In general, the concentrations of heavy metals in the water samples at the research locations were similar to previous studies, though the levels of Fe and Zn were categorized as high in this research.

Bazzi (2014) stated that anthropogenic activity is a determining factor for high Zn and Fe metal contamination in waters. ^51^ Furthermore, Sun *et al.* (2020) emphasized that heavy metal levels are influenced by changes in salinity, pH, and biological activity, they tend to increase at the location of bays and estuaries that are close to residential activities. ^52^ Zn and Fe metal contamination in the research location is influenced by community activities, especially settlements that produce various types of waste. In addition, the open land of former bauxite mining on Bintan Island is a source of contamination of these metals in the waters. According to Rezaei *et al.* (2019) and Ismail *et al.* (2019), bauxite waste is a source of Fe and Zn metal contamination in the environment. ^53, 54^ It is one of the causes of the high concentration of Zn and Fe metals in the research location.

### Heavy metals in sediment samples

Heavy metals that enter the water accumulate at the bottom and affect the metal composition of the sediments. According to preliminary studies, the highest content of heavy metal Cd in sediments occurred in the westerly monsoon with an average of 0.0289 ± 0.037 μg/g, while the northerly monsoon produced strong current dynamics of 0.0045 ± 0.001 μg/g. The highest Pb content was in the easterly monsoon with an average of 0.0449 ± 0.013 μg/g, the highest Cu and Fe was in the west season with an average of 0.0321 ± 0.024 μg/g and 18.43 ± 5.25 μg/g, respectively. However, the heavy metal Zn actually increased during the northerly monsoon with an average value of 11.63 ± 14.041 μg/g, as shown in Table 5.

**Table 5.** Average concentration of heavy metal (μg/g) of Cd, Pb, Cu, Fe, and Zn in the sediment during Northerly–Westerly monsoon in Bintan Island coastal water. Cd = cadmium, Pb = lead, Cu = copper, Fe = iron, Zn = zinc.

Season	Location	Cd	Pb	Cu	Fe	Zn
*Northerly monsoon*	Malang Rapat	0.0029±0.0013	0.0504±0.015	0.0064±0.015	18.06±6.08	0.747±15.06
	Teluk Bakau	0.0056±0.0007	0.0327±0.0151	0.0371±0.014	14.28±6.64	26.61±15.3
	Beralas Pasir	0.0041±0.0006	0.0626±0.0145	0.0161±0.007	26.19±7.53	27.06±15.1
	Berakit	0.0053±0.001	0.0429±0.0063	0.0083±0.007	15.13±9.05	0.34±14.97
	Pengudang	0.0049±0.001	0.0341±0.0075	0.0230±0.007	11.80±9.36	26.25±14.97
	Sakera	0.0034±0.0011	0.0464±0.007	0.0109±0.0038	28.89±7.79	0.295±6.59
	Tg. Siambang	0.0054	0.0326±0.0093	0.008±0.0051	13.7±2.33	0.1281±6.33
	***Average***	**0.0045±0.001**	**0.0431±0.011**	**0.0157±0.011**	**18.29±6.63**	**11.63±14.041**
*Easterly monsoon*	Malang Rapat	0.0031±0.002	0.0512±0.017	0.0061±0.012	15.25±3.54	1.283±14.02
	Teluk Bakau	0.0072±0.0019	0.0231±0.021	0.0298±0.011	15.38±3.91	17.30±14.05
	Beralas Pasir	0.0034±0.001	0.0541±0.014	0.0261±0.010	21.45±5.48	29.24±15.95
	Berakit	0.0050±0.0009	0.0623±0.0146	0.0092±0.01	14.14±9.73	1.24±15.99
	Pengudang	0.0031±0.0014	0.0349±0.007	0.0277±0.01	10.72±9.71	28.49±16.29
	Sakera	0.0042±0.0008	0.0394±0.004	0.0251±0.08	29.03±7.83	0.34±6.28
	Tg. Siambang	0.0059±0.0039	0.0491±0.008	0.0092±0.06	14.22±1.5	0.193±2.83
	***Average***	**0.0046±0.0015**	**0.0449±0.013**	**0.0190±0.0103**	**17.17±6.12**	**11.16±13.52**
*Southerly monsoon*	Malang Rapat	0.012±0.048	0.0612±0.023	0.0058±0.029	16.39±0.52	2.195±12.27
	Teluk Bakau	0.0920±0.05	0.0170±0.031	0.0117±0.027	17.21±4.38	9.82±11.28
	Beralas Pasir	0.0041±0.0011	0.0271±0.025	0.0594±0.023	16.22±3.67	26.21±11.06
	Berakit	0.0051±0.0013	0.0762±0.026	0.0117±0.018	9.17±11.38	4.59±9.7
	Pengudang	0.0029±0.0015	0.0431±0.009	0.0390±0.020	14.52±9.31	19.48±10.46
	Sakera	0.0054±0.0072	0.0249±0.008	0.0463±0.019	31.01±8.6	1.24±4.57
	Tg. Siambang	0.0058±0.034	0.0386±0.018	0.0083±0.01	15.27±2.15	1.482±4.39
	***Average***	**0.0182±0.032**	**0.0412±0.021**	**0.0260±0.021**	**17.11±6.67**	**9.29±9.904**
*Westerly monsoon*	Malang Rapat	0.071±0.036	0.0712±0.029	0.0082±0.009	12.82±4.5	1.87±10.05
	Teluk Bakau	0.0921±0.045	0.0121±0.016	0.0162±0.035	19.87±4.45	9.26±8.12
	Beralas Pasir	0.0210±0.009	0.0411±0.04	0.0262±0.033	21.21±4.3	21.76±7.8
	Berakit	0.0065±0.002	0.0121±0.042	0.0825±0.029	12.92±7.54	6.53±7.82
	Pengudang	0.0021±0.002	0.0922±0.038	0.0245±0.010	18.27±6.24	17.23±8.87
	Sakera	0.0072±0.014	0.0262±0.008	0.0425±0.009	27.82±6.21	1.982±3.9
	Tg. Siambang	0.0022±0.013	0.0255±0.009	0.0244±0.004	16.07±1.21	1.726±4.49
	***Average***	**0.0289±0.037**	**0.0401±0.031**	**0.0321±0.024**	**18.43±5.25**	**8.62±8.049**

* The residential areas: Malang Rapat, Teluk Bakau, Beralas Pasir, Pengudang, Sakera.

** The ex-bauxsite mine: Tg. Siambang.

The concentration of heavy metals in sediments decreases and increases in the northern monsoon and east to west monsoons, respectively. The stirring of sediment that occurs in the northern monsoon is a factor in the difference in metal concentrations between seasons. The water currents stir the sediment in seasons with hydrodynamic conditions, therefore, the heavy metals contained are dispersed more quickly. Meanwhile, in other seasons, the dynamics are smaller and support the deposition of metal particles that enter the waters. This affects the accumulation of heavy metals in the water to the bottom sediments. Wardani *et al.* (2020) stated that heavy metals are transported by currents where the flow conditions differ in strength in accordance with the season. ^55^ Wisha *et al.* (2018) and Hamuna and Tanjung (2021) stated that the distribution of heavy metals in the sea is influenced by several water inputs and dynamics, topography, wind patterns, movement and surface current circulation. ^56, 57^

## Conclusions

In conclusion, the concentration of Cd, Pb, Cu, Fe and Zn in *C. racemosa* ranged from 0.14–4.83 μg/g, 1.07–47.95 μg/g, 1.14–8.14 μg/g, 62.4–609.7 μg/g, and 12.7–76.5 μg/g with averages of 0.95 ± 1.15 μg/g, 14.17 ± 10.98 μg/g, 3.07 ± 2.16 μg/g, 220.58 ± 175.16 μg/g, and 35.25 ± 16.54 μg/g, respectively. Heavy metal fluctuations are closely related to seasonal changes, where in the east-west monsoon (Easterly–Westerly monsoon), those that accumulate in *C. racemosa* are lower than in the north monsoon (Northerly monsoon). However, based on the one-way Anova analysis, only Pb was significantly different between seasons, while Cd, Cu, Fe, and Zn were insignificant. The correlation between metals showed a positive relationship, which indicates that the increase in one metal affect others.

## Data availability

All data underlying the results are available as part of the article and no additional source data are required.
