# Supplementary material for: Accumulation of essential (copper, iron, zinc) and non-essential (lead, cadmium) heavy metals in Caulerpa racemosa, sea water, and marine sediments of Bintan Island, Indonesia
Source: F1000Res. 2022 Feb 1;10:699. Originally published 2021 Jul 30. [Version 2] doi: 10.12688/f1000research.54445.2 (PMC8894816; doi:10.12688/f1000research.54445.2)
Supplement: Peer review history

# Peer review: Reviewer response for version 2

**DOI:** 10.5256/f1000research.120569.r126138

**Published:** 2022-03-03

**Authors:** Zainal Abidin Muchlisin

**Affiliations:** 1 Faculty of Marine and Fisheries, Universitas Syiah Kuala, Banda Aceh, Indonesia

Title: I think the title should be rewritten to be short and clear.

 Abstract: The author has to provide a clear objective(s) in the abstract. Conclusion in the abstract is also unclear, please refer to the objective(s). For example, you said that "The levels of these heavy metals in the sediment are also high....". But how much higher, or what standard you used, is still not clear.

 Keywords: Some keywords are not specific and too general. 

 Introduction: The problem statement and state of the arts of the study are unclear. The author must clearly state what previous studies have done to solve the current problem, what aspects have never been explored/studied and why these aspects are important etc. What is the specific problem in these waters, and also why did you use *C. racemosa*? Why not other seaweed? The objective of the study must be clear.

 Methods: I think the methods section is acceptable, but maybe can be more detailed, especially on the seaweed sampling, and also water and sediment.

 Results: I think would be better if the results were separated from the discussion. In general, the presentation of the results is acceptable.

 Discussion: The discussion is still shallow and still needs deeper analysis and synthesis by comparing the results of the latest related studies.

 Conclusion: The conclusion is too wide and not focused to answer the objective(s).

 References: Please add more updated references from reputable journals.

Is the work clearly and accurately presented and does it cite the current literature?

Partly

If applicable, is the statistical analysis and its interpretation appropriate?

Yes

Are all the source data underlying the results available to ensure full reproducibility?

Yes

Is the study design appropriate and is the work technically sound?

Yes

Are the conclusions drawn adequately supported by the results?

Yes

Are sufficient details of methods and analysis provided to allow replication by others?

Yes

Reviewer Expertise:

NA

I confirm that I have read this submission and believe that I have an appropriate level of expertise to confirm that it is of an acceptable scientific standard.

# Peer review: Reviewer response for version 2

**DOI:** 10.5256/f1000research.120569.r121801

**Published:** 2022-02-22

**Authors:** Ong Meng Chuan

**Affiliations:** 1 Faculty of Science and Marine Environment, Universiti Malaysia Terengganu, Terengganu, Malaysia

Author has revised the manuscript according to the comments. In my opinion, this manuscript can be indexed.

Is the work clearly and accurately presented and does it cite the current literature?

Partly

If applicable, is the statistical analysis and its interpretation appropriate?

No

Are all the source data underlying the results available to ensure full reproducibility?

Partly

Is the study design appropriate and is the work technically sound?

Partly

Are the conclusions drawn adequately supported by the results?

No

Are sufficient details of methods and analysis provided to allow replication by others?

Partly

Reviewer Expertise:

marine pollution; heavy metals pollution

I confirm that I have read this submission and believe that I have an appropriate level of expertise to confirm that it is of an acceptable scientific standard.

# Response

**Published:** 2022-02-23

**Authors:** hilfi pardi

Dear Prof. Ong Meng Chuan

 Thank you for reviewing and providing constructive feedback for our article.

# Peer review: Reviewer response for version 1

**DOI:** 10.5256/f1000research.57932.r99666

**Published:** 2021-11-17

**Authors:** Ong Meng Chuan

**Affiliations:** 1 Faculty of Science and Marine Environment, Universiti Malaysia Terengganu, Terengganu, Malaysia

**Methods:**

- Provide citation for the acid digestion used in this study.
- What standard reference material was used in this study? What is the recovery test?
- No citation provided for the water analysis and sediment analysis.
- No detailed digestion method provided.

**Results & discussion:**

- I would suggest that the data is presented in graphs for the comparison between.
- I'm not sure whether the data presented is acceptable or not since no recovery percentage is provided.
- Are the authors Khaled *et al.* also studying the same sp - because the authors only mention macroalgae. 
- More explanation about why the ex-bauxite mining area is high with metals content.
- For the correlation of metals, the authors only compare the data with other investigators - should include what the correlation means? Any correlation with size?
- The concentration of metals in sediment is very low. But I can't say whether the data is acceptable or not, since no recovery percentage is provided. Please check the data.
- Since the authors study on organisms, water and sediment, what is the correlation between these three samples? No explanation is provided. Suggest to run PCA analysis.

**Conclusion:**

- The conclusion is only based on organisms - how about water and sediment? Please rewrite.

Is the work clearly and accurately presented and does it cite the current literature?

Partly

If applicable, is the statistical analysis and its interpretation appropriate?

No

Are all the source data underlying the results available to ensure full reproducibility?

Partly

Is the study design appropriate and is the work technically sound?

Partly

Are the conclusions drawn adequately supported by the results?

No

Are sufficient details of methods and analysis provided to allow replication by others?

Partly

Reviewer Expertise:

marine pollution; heavy metals pollution

I confirm that I have read this submission and believe that I have an appropriate level of expertise to confirm that it is of an acceptable scientific standard, however I have significant reservations, as outlined above.

# Response

**Published:** 2022-01-15

**Authors:** hilfi pardi

1. Provide citation for the acid digestion used in this study.
  Response: 
  Added: V. van Ginneken and E. de Vries, Seaweeds as Biomonitoring System for Heavy Metal (HM) Accumulation and Contamination of Our Oceans, Am. J. Plant Science. 09 (2018), pp. 1514–1530.
2. The analytical procedures must be validated, one of which is using the recovery test
  Response:
  The analytical procedures must be validated, one of which is using the recovery test
3. No citation provided for the water analysis and sediment analysis.
  Response:
  S. Shanbehzadeh, M. Vahid Dastjerdi, A. Hassanzadeh and T. Kiyanizadeh, *Heavy metals in water and sediment: A case study of Tembi River*, J. Environ. Public Health 2014 (2014), .
4. No detailed digestion method provided.
  Response:
  It can be seen in the following references:
  V. van Ginneken and E. de Vries, *Seaweeds as Biomonitoring System for Heavy Metal (HM) Accumulation and Contamination of Our Oceans*, Am. J. Plant Sci. 09 (2018), pp. 1514–1530.
5. For the correlation of metals, the authors only compare the data with other investigators - should include what the correlation means? Any correlation with size?
  Response:
  In this study, the PCA test and the correlation between heavy metals contained in water, *Caulerpa*, and sediment were carried out with water quality parameters.
6. References Khaled et al. (2014) less relevant
  Response:
  Modified and refers to Ginneken and Vries (2018).
7. Why is the content of heavy metals in the ex-bauxite area higher?
  Response:
  It is explained in the chapter on the results and discussion of the sub-chapter “Heavy metals in *Caulerva racemosa* samples” the 5th paragraph.
8. Since the authors study on organisms, water and sediment, what is the correlation between these three samples? No explanation is provided. Suggest to run PCA analysis.
  Response:
  Added a new chapter “PCa and Correlation of heavy metal” in the results and discussion chapter.
9. Correlation Test Results:
  - Between water quality and metal concentration in Cauleroa
  - Between water quality and metal concentration in water
  - Between water quality and metal concentration in sediment
  Response:
  Listed in Tables 4, 5, and 6 of the Appendix.
10. I would suggest that the data is presented in graphs for the comparison between.
  Response:
  Results are listed in Figures 3, 4 and 5.
11. Pca analysis includes the scatterplot
  Response:
  Results listed on Figure 6.
12. The conclusion is only based on organisms - how about water and sediment? Please rewrite.
  Response:
  Was added to the conclusion for metals in water and sediment.
